# Predicting prognosis using stroke-heart indicator: brain natriuretic peptide in patients with aneurysmal subarachnoid hemorrhage

**DOI:** 10.3389/fneur.2025.1510235

**Published:** 2025-01-22

**Authors:** Jionghao Xue, Fa Lin, Minghao Liu, Wenxiong Song, Runting Li, Yu Chen, Jun Yang, Heze Han, Yitong Jia, Xiaolin Chen, Rong Wang, Yuanli Zhao

**Affiliations:** ^1^Department of Neurosurgery, Beijing Tiantan Hospital, Capital Medical University, Beijing, China; ^2^China National Clinical Research Center for Neurological Discases, Beijing, China; ^3^Department of Neurosurgery, Peking Union Medical College Hospital, Chinese Academy of Medical Sciences and Peking Union Medical College, Beijing, China

**Keywords:** stroke heart disease, ASAH, bnp, retrospective studies, risk factors

## Abstract

**Objective:**

This study aims to explore the correlation between brain natriuretic peptide (BNP) levels and prognosis in patients suffering from aneurysmal subarachnoid hemorrhage (aSAH).

**Methods:**

This retrospective study included patients diagnosed with aneurysmal subarachnoid hemorrhage (aSAH) at Beijing Tiantan Hospital between January 2015 and September 2021. Plasma BNP levels were measured upon admission and log-transformed to reduce skewness. Elevated BNP was defined as lgBNP ≥1.79 (equivalent to BNP ≥62 pg./mL). The primary outcome was poor prognosis, defined as a modified Rankin Scale (mRS) score ≥ 3 at 90 days. Univariable and multivariable logistic regression analyses were conducted to examine the association between BNP levels and prognosis. Additionally, we assessed the potential impact of incorporating BNP into a predictive model for poor prognosis.

**Results:**

The statistical analysis encompassed a total of 932 patients. Among them, 171 individuals experienced unfavorable prognosis (mRS ≥3) during follow-up, and 444 patients had elevated BNP levels, defined as lgBNP ≥1.79. After accounting for confounding factors, elevated BNP levels remained a significant independent risk factor of a poor prognosis (*p* = 0.047, OR = 1.49, 95%CI = 1.01–2.20). Nevertheless, BNP’s predictive value alone might not warrant its inclusion in a prognostic model.

**Conclusion:**

Elevated BNP levels independently forecast unfavorable prognosis in patients with aSAH, even though the cutoff value is lower than the cardiology standards. Continuous monitoring and personalized hospitalization plans can be vital for these patients.

## Introduction

Aneurysmal subarachnoid hemorrhage (aSAH) is a common neurosurgical condition characterized by the rupture of cerebrovascular structures, leading to bleeding into the subarachnoid space. According to a survey conducted by the World Health Organization (WHO), the annual incidence of aSAH in China is reported to be 2.0 per 100,000 population (1).

aSAH not only inflicts significant pain on patients but also carries a high mortality rate, ranging from 32 to 67%. Furthermore, one-third of survivors are left with physical disabilities (2). Additionally, aSAH can give rise to various complications, including vasospasm, delayed cerebral ischemia (DCI), and hydrocephalus (3–5). Given these factors, it is crucial to assess the risk of poor prognosis during hospitalization and identify patients who require intensive treatment. Currently, widely employed evaluation methods involve the use of various scoring scales. While these scales provide a quantitative assessment of patients’ condition, they have certain limitations in practical application. For instance, patients with aSAH may exhibit varying degrees of consciousness disorders, which can influence the scoring process. Moreover, symptom-based scoring systems rely on the subjective judgment of physicians, leading to potential discrepancies among different raters. Conducting systematic scoring is time-consuming and may be challenging for larger medical centers, and it could also cause dissatisfaction among patients. Therefore, it is important to identify more convenient and direct indicators for evaluating patients’ condition and prognosis.

B-type natriuretic peptide (BNP) is a vasodilatory peptide secreted by ventricular cardiomyocytes and hypothalamus (6). It is released in response to cardiomyocyte stretching caused by elevated filling pressure, and it plays a role in regulating blood pressure and fluid balance. BNP has been recognized as a biomarker for cardiac dysfunction (7, 8). It is worth noting that many patients with neurological injuries also experience secondary cardiac injury (9). As a biomarker for cardiac dysfunction, BNP has been found to be elevated in the acute phase of traumatic brain injury, and it has been associated with intracranial pressure (ICP), hydrocephalus, and stroke severity (10, 11).

Some researchers have explored the predictive value of cardiac biomarkers, such as cardiac troponin I (cTnI), for the prognosis of patients with aSAH and ischemic stroke (12, 13). As for BNP, recent studies have shown that BNP is independently associated with short-term outcomes following the onset of ischemic stroke (14, 15). Furthermore, there have been suggestions regarding the possibility of BNP being a novel risk factor of early postoperative seizures (16).

Previous studies investigating the relationship between BNP and outcomes in aSAH have been limited by smaller sample sizes. In addition, they mainly focus on outcome events such as cerebral infarction, delayed cerebral ischemia (DCI), or mortality (17–19). Our study, with a larger cohort, shifts the focus toward functional outcomes, as assessed by the modified Rankin Scale (mRS), providing a more comprehensive evaluation of patient prognosis and long-term neurological recovery.

## Methods

### Study population

**Figure 1:**
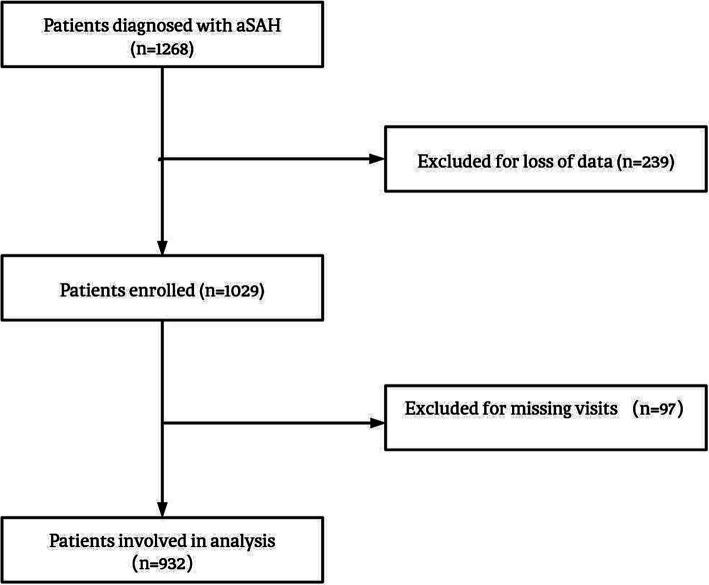
Study flowchart. aSAH, aneurysmal subarachnoid hemorrhage.

We collected data from aSAH patients who were hospitalized in Beijing Tiantan Hospital between January 2015 and September 2021. The inclusion criteria for the study were as follows: (1) Age of 18 years or older; (2) Confirmed diagnosis of aSAH through computed tomography, lumbar puncture, or magnetic resonance imaging; (3) Signed informed consent. The main exclusion criteria were as follows: (1) physical disability due to any previous disease; (2) treatment including external ventricular drainage, lumbar puncture, angiography, intubation, and/or mechanical ventilation at other hospitals before presentation to our hospital; and (3) missing data, including medical, radiological, and laboratory information. A total of 932 patients were included in the analysis. The process of patient selection is illustrated in Figure 1, illustrating the study’s flowchart. All patients included in this analysis were participants in the Long-term Prognosis of Emergency Aneurysmal Subarachnoid Hemorrhage (LongTEAM) Registry study, with registration number NCT 04785976.

### Ethical standards

This study was approved by the ethics committee of Beijing Tiantan Hospital. All participants or their legal representatives have duly endorsed informed consent. The hospitalization procedures were meticulously established in strict adherence to the prescribed guidelines (20).

### Clinical variables

All the data used in this study were obtained from patients’ medical records. The baseline data included the following variables: age, gender, hypertension, hyperlipemia, diabetes mellitus, hydrocephalus, admission coma or seizure, and various neurological scores including Graeb score, subarachnoid hemorrhage early brain edema (SEBES) score, modified Fisher scale (mFS) grade, World Federation of Neurological Surgeons (WFNS) grade, Hunt-Hess (H-H) grade, mRS. The treatment modalities were categorized as surgery and microscopic clipping/endovascular coiling. The neurological scores were transformed into categorical variables based on the following criteria: Graeb score of 5–12, SEBES score of 3–4, mFS grade of 3–4, WFNS grade of 4–5, H-H grade of 4–5, and mRS score of 3–6 were defined as indicators of poor condition, respectively.

### Outcome measures

All the patients received follow-up via telephone or outpatient 90 days after discharge. 3-month mRS 3–6 was defined as poor prognosis. Complication such as delayed cerebral ischemia (DCI) defined as reduction in blood flow to brain that occurs after a significant brain injury was documented too. BNP was measured routinely at the time of emergency admission for all aSAH patients as part of standard clinical practice.

### Statistic analysis

All analyses were performed using SPSS Statistics 26.0 (IBM, Armonk, New York, United States) and R (version 2.12.2). Two-tailed *p* values <0.05 were considered statistically significant. Descriptive variables were summarized as mean ± SD or median with interquartile range for continuous variables, and categorical variables were summarized as frequencies (percentage). Student’s t-test or the Mann–Whitney test were used to compare the quantitative variables, and The χ^2^ test or Fisher’s exact tests were used within categorical variables.

We examined the factors associated with elevated BNP to identify which variables need adjustment in the multivariable regression model and assessed the logical coherence between variables, thereby enhancing the credibility of the analysis (21).

To build a regression model and evaluate the predictive value of BNP, both uni-and multivariate analysis were performed to evaluate the influence of each variable on exceeded 90-day mRS score. In the univariable analysis, we included clinical factors commonly associated with outcomes in aSAH, based on prior research and clinical relevance. These factors were selected *a priori* to ensure comprehensive coverage of potential confounders. For the multivariable analysis, significant variables from the univariable analysis (*p* < 0.05) were included in a stepwise logistic regression model. In addition to variables that were significant in univariable analysis (*p* < 0.05), certain factors, such as sex, were included in the multivariable analysis based on their established clinical relevance and prior evidence from the literature. This approach ensures that potential confounders with known prognostic importance are adequately controlled for, even if their univariable association with the outcome was not statistically significant. We employed a stepwise logistic regression approach (Likelihood Ratio method) to construct the final multivariable model. This method iteratively selects variables based on their contribution to model fit, excluding those that are nonsignificant in the multivariable context.

To compare the discriminatory performance of the predictive models, we used the DeLong test to evaluate whether the inclusion of BNP in the model resulted in a significant improvement in the area under the receiver operating characteristic (AUC). Additionally, to further assess the clinical utility of BNP in the predictive model, we performed the Net Reclassification Improvement (NRI) test. The NRI quantifies the improvement in the reclassification of patients into more accurate risk categories with the inclusion of BNP. This test is particularly useful for capturing changes in risk classification, even in cases where the AUC difference may be modest.

To mitigate issues of multicollinearity and heteroscedasticity and transform the data to approximate a normal distribution logarithmic transformation was performed for BNP. We created a receiver operating characteristic (ROC) curve to assess the relationship between BNP and admission mRS, and determined the cutoff value using Youden’s index. Additionally, we also used the cardiology standards (lgBNP≥2 vs. <2) to explore the relationship between BNP and the 3-month mRS score.

## Results

### Baseline characteristics

A total of 932 patients with aSAH were recruited in our study, their baseline characteristics are shown as Table 1. Among the patients, 553 (59.3%) were female. The mean age was 55.4 ± 11.3 years old. 486 (52.1%) accepted surgical treatment, and the rest accepted interventional therapy. 36 (3.9%) suffered loss of consciousness and seizure. At admission, 377 (40.4%) patients’ nervous system were in bad condition (mRS 3–6), and 172 (18.4%) had higher mRS in our follow-up, which means poor prognosis. After calculating Youden’s index, lgBNP ≥1.79 was defined as indicative of elevated BNP and 444 (47.6%) patients’ BNP exceeded this standard.

**Table 1:** Patients’ baseline characteristics.

Characteristic	Statistics
Age, years(mean ± SD)	55.4 ± 11.3
Female, n (%)	553 (59.3)
Graeb score 5–12, n (%)	70 (7.5)
SEBES score 3–4, n (%)	439 (47.1)
mFS grade 3–4, n (%)	648 (69.5)
WFNS grade 4–5, n (%)	191 (20.5)
H-H grade 4–5, n (%)	81 (8.7)
Surgery, n (%)	486 (52.1)
Hypertension, n (%)	531 (56.9)
Hyperlipemia, n (%)	71 (7.6)
Diabetes mellitus, n (%)	71 (7.6)
Hydrocephalus, n (%)	361 (38.7)
coma+seizure, n (%)	36 (3.9)
lgBNP≥1.79, n (%)	444 (47.6)
Admission mRS 3–6, n (%)	377 (40.4)
3-months mRS 3–6, n (%)	172 (18.4)

SD, standard; SEBES, subarachnoid hemorrhage early brain edema; mFS, modified Fisher scale; WFNS, World Federation of Neurological Surgeons; H-H, Hunt-Hess; BNP, brain natriuretic peptide.

### Association between BNP and other variables

In univariate analyses, there were significant associations between elevated BNP and age, gender, hydrocephalus, DCI and worse nervous system score such as Graeb, mFS, WFNS and H-H grade. After adjustment for confounding factors, age, gender, mFS, WFNS and DCI still remain the significant association (Table 2). The abnormal increase of BNP shows similar tendency with neurological score. Patients with elevated BNP have more risk suffering from DCI. The association between elevated BNP and increased risk of DCI was confirmed in the multivariable logistic regression model (OR 1.61, 95% CI: 1.19–2.18, *p* = 0.002).

**Table 2:** Association between BNP and other variables.

Variables	Univariate				Multivariate	
	Elevated BNP	Normal BNP	*p*	OR (95%CI)	*p*	OR (95%CI)
Number (N)	444	488				
Age, years(mean ± SD)	57.2 ± 11.4	53.8 ± 11.0	<0.001	—	0.001	1.02 (1.01–1.03)
Female, n (%)	289 (65.1)	264 (54.1)	0.001	0.63 (0.49–0.82)	0.004	0.66 (0.50–0.88)
Graeb score 5–12, n (%)	47 (10.6)	23 (4.7)	0.001	2.39 (1.43–4.01)		
SEBES score 3–4, n (%)	221 (49.8)	217 (44.5)	0.105	1.24 (0.96–1.60)		
mFS grade 3–4, n (%)	334 (75.2)	313 (64.1)	<0.001	1.70 (1.28–2.26)	0.030	1.39 (1.03–1.88)
WFNS grade 4–5, n (%)	120 (27.0)	70 (14.3)	<0.001	2.21 (1.59–3.07)	0.001	1.77 (1.24–2.51)
H-H grade 4–5, n (%)	50 (11.3)	30 (6.1)	0.005	1.94 (1.21–3.11)		
Hypertension, n (%)	264 (59.5)	266 (54.5)	0.127	1.22 (0.94–1.59)		
Hyperlipemia, n (%)	27 (6.1)	44 (9.0)	0.092	0.65 (0.40–1.07)		
Diabetes mellitus, n (%)	35 (7.9)	36 (7.4)	0.771	1.07 (0.66–1.74)		
Surgery, n (%)	228 (51.4)	257 (52.7)	0.689	0.95 (0.73–1.23)		
Hydrocephalus, n (%)	188 (42.3)	173 (35.5)	0.031	1.34 (1.03–1.74)		
coma+seizure, n (%)	18 (4.1)	17 (3.5)	0.647	1.17 (0.60–2.30)		
DCI, n (%)	107 (21.9)	153 (34.5)	<0.001	1.87 (1.40–2.50)	0.002	1.61 (1.19–2.18)

SD, standard; SEBES, subarachnoid hemorrhage early brain edema; mFS, modified Fisher scale; WFNS, World Federation of Neurological Surgeons; H-H, Hunt-Hess; BNP, brain natriuretic peptide; DCI, delayed cerebral ischemia. *p* < 0.05 was accepted as significant.

### Risk factors of poor prognosis

In our follow-up, 171 patients’ mRS score ranged from 3 to 6. In univariate analysis, higher mRS showed significant association with higher age, surgical treatment, hypertension, hydrocephalus, elevated BNP and higher nervous system score including Graeb, mFS, WFNS and H-H. After adjusting for age, sex, hypertension, hydrocephalus, the forward stepwise multivariate analysis showed that age (*p* < 0.001, OR = 1.07, 95%CI = 1.05–1.09), higher Graeb score (*p* = 0.004, OR = 2.46, 95%CI = 1.33–4.55), higher WFNS grade (*p* < 0.001, OR = 3.38, 95%CI = 2.06–5.52), higher H-H grade (*p* = 0.005, OR = 2.56, 95%CI = 1.34–4.91), surgical treatment (*p* < 0.001, OR = 2.77, 95%CI = 1.84–4.17), higher BNP (*p* = 0.047, OR = 1.49, 95%CI = 1.01–2.20) were independent risk factors of poor prognosis (Table 3). In the univariable analysis, mFS grade (*p* < 0.001), Hypertension (*p* = 0.001), Hydrocephalus (*p* = 0.017), and lgBNP≥2 (*p* = 0.001) were identified as significant. However, these variables were excluded during stepwise selection due to their lack of independent significance in the multivariable context.

**Table 3:** Risk factors of poor prognosis (3-months mRS).

Variables	Univariate				Multivariate	
	3 m-mRS 3–6	3 m-mRS 0–2	*p*	OR (95%CI)	*p*	OR (95%CI)
Number (N)	171	761				
Age, years(mean ± SD)	60.9 ± 10.7	54.1 ± 11.0	<0.001		<0.001	1.07 (1.05–1.09)
Female, n (%)	102 (59.6)	451 (59.3)	0.926	0.98 (0.70–1.38)		
Graeb score 5–12, n (%)	32 (18.7)	38 (5.0)	<0.001	4.38 (2.65–7.25)	0.004	2.46 (1.33–4.55)
SEBES score 3–4, n (%)	82 (48.0)	356 (46.8)	0.781	1.05 (0.75–1.46)		
mFS grade 3–4, n (%)	142 (83.0)	505 (66.4)	<0.001	2.48 (1.62–3.80)		
WFNS grade 4–5, n (%)	85 (49.7)	105 (13.8)	<0.001	6.18 (4.29–8.88)	<0.001	3.38 (2.06–5.52)
H-H grade 4–5, n (%)	49 (28.7)	31 (4.1)	<0.001	9.46 (5.80–15.42)	0.005	2.56 (1.34–4.91)
Surgery, n (%)	112 (65.5)	373 (49.0)	<0.001	1.98 (1.40–2.79)	<0.001	2.77 (1.84–4.17)
Hypertension, n (%)	117 (68.4)	413 (54.3)	0.001	1.83 (1.28–2.60)		
Hyperlipemia, n (%)	12 (7.0)	59 (7.8)	0.743	0.90 (0.47–1.71)		
Diabetes mellitus, n (%)	18 (10.5)	53 (7.0)	0.113	1.57 (0.90–2.76)		
Hydrocephalus, n (%)	80 (46.8)	281 (36.9)	0.017	1.50 (1.08–2.10)		
coma+seizure, n (%)	7 (4.1)	28 (3.7)	0.797	1.12 (0.48–2.60)		
lgBNP**≥**1.79, n (%)	107 (62.6)	337 (44.3)	<0.001	2.10 (1.50–2.96)	0.047	1.49 (1.01–2.20)
lgBNP**≥**2, n (%)	72 (42.1)	218 (28.6)	0.001	1.81 (1.29–2.55)		

SD, standard; SEBES, subarachnoid hemorrhage early brain edema; mFS, modified Fisher scale; WFNS, World Federation of Neurological Surgeons; H-H, Hunt-Hess; BNP, brain natriuretic peptide. *p* < 0.05 was accepted as significant.

**Figure 2:**
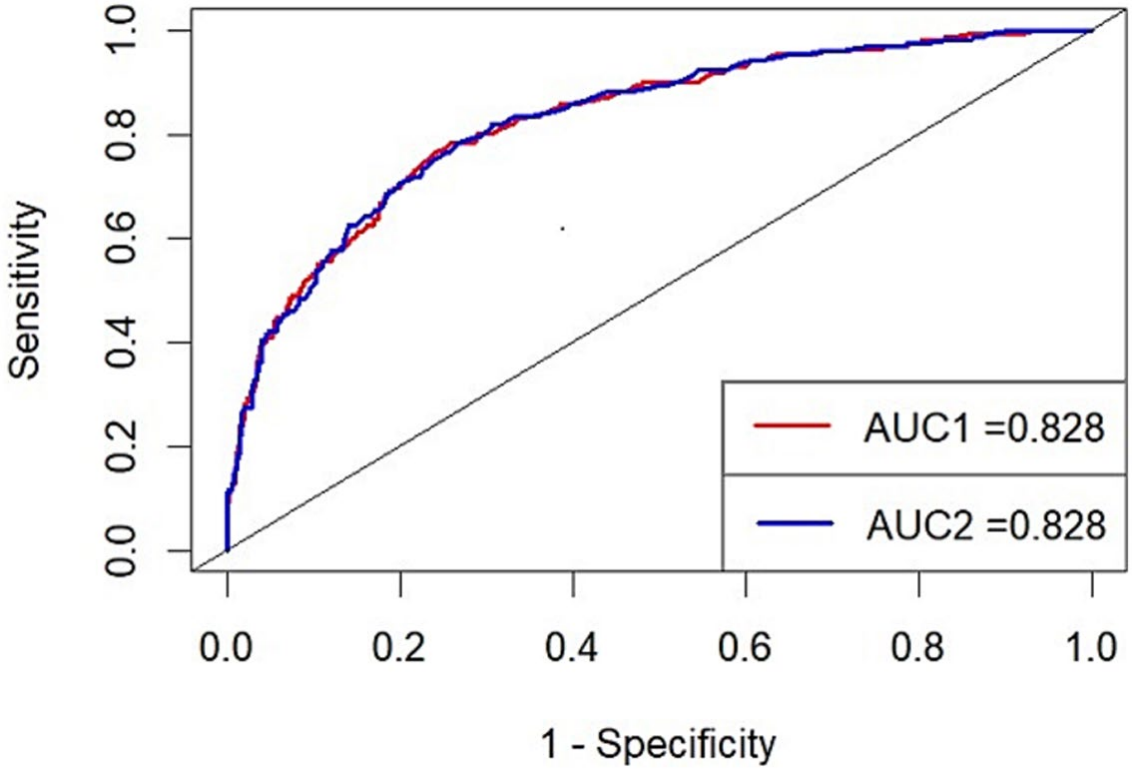
ROC1 (red): the predict model with BNP (Variables included: age, WFNS grade, sugery, BNP, Graeb score, H-H grade) ROC2 (blue): the predict model without BNP (Variables included: age, WFNS grade, sugery, Graeb score, H-H grade) AUC, area under the curve.

We enrolled these independent risk factors in a model and drew its ROC and calculated the AUC (AUC1; Figure 2). The AUC1 is 0.828, which means the model can play a role in predicting poor prognosis. To evaluate if elevated BNP deserves being added in predict model of poor prognosis, we built another model excluding BNP and calculated its AUC (AUC2; Figure 2). The two AUC showed no differences (AUC1 0.828, 95% CI 0.793–0.862 vs. AUC2 0.828, 95% CI 0.794–0.862). The DeLong test showed that the inclusion of BNP did not result in a statistically significant improvement in AUC (*p* = 0.596), indicating that BNP did not substantially enhance the model’s discriminatory ability. The NRI value was 0.0111 (*p* = 0.401), suggesting that the inclusion of BNP led to a modest, though statistically non-significant, improvement in risk reclassification. It seems that BNP does not have enough value to be added in the predict model of poor prognosis.

## Discussion

In our study, we discovered that age, gender, and certain rating scales (with the exception of SEBES) were associated with BNP levels. After adjusting for these variables, BNP remained an independent risk factor of the 3-month modified Rankin Scale (mRS) score. Similar findings have been observed in the studies conducted by James and Amber (22, 23). However, the former study only reported the association in univariate analysis, and the latter study used cTnI instead of mRS as the outcome indicator, which may not accurately reflect the association between BNP and patient outcomes. Additionally, both studies had relatively small sample sizes. Hideyuki Kishima conducted a retrospective study with a larger sample size compared to previous studies. However, the outcome indicator in their study was limited to death, and the survivors with poor prognosis were not taken into account (19).

Compared with existing literature, which often centers on cerebral infarction, DCI, or mortality as primary outcomes, our study provides a novel perspective by emphasizing functional outcomes represented by the mRS score. This approach not only aligns more closely with the real-world challenges of post-aSAH management but also offers a more nuanced understanding of the long-term impact of elevated BNP levels on patient prognosis. Additionally, the larger sample size in our study strengthens the robustness and generalizability of the findings. The department of cardiology has defined a normal BNP level as BNP < 100. In our study, we found that lgBNP >1.79 (corresponding to BNP ≥ 62 pg./mL) predicted poor prognosis more effectively than lgBNP >2 (corresponding to BNP ≥ 100 pg./mL). Although the risk of cardiac events is low in patients with BNP levels between 61.7 and 100, they still carry a risk of poor prognosis in the nervous system. This suggests the possibility that BNP directly influences the prognosis of the neurological system.

Although DCI is associated with elevated BNP and poor prognosis, it was excluded from the predictive model to avoid introducing mediators that might obscure direct predictive relationships. Additionally, BNP serves as an early biomarker, while DCI typically manifests later in the clinical course, limiting its utility for early prognosis.

Cardiovascular disease and cerebrovascular disease pose significant threats to human health. In the past, these two conditions were often considered as separate systems in medical practice. However, with advancements in medical knowledge and our evolving understanding of the spectrum of human diseases, the concept of brain-heart comorbidity has gained recognition. In the study conducted by Prosser, it was found that approximately 20% of patients with ischemic stroke experienced serious adverse cardiac events, particularly within the first 3 days after the stroke (24). More recent studies, both in animals and clinical settings, have demonstrated that similar cardiac events can occur in other acute brain diseases such as hemorrhagic stroke and traumatic brain injury, suggesting the presence of shared mechanisms (25). A review by Jan F Scheitz in 2018 introduced the concept of stroke-heart syndrome, which refers to cardiac damage secondary to neurological diseases (26). Stroke-heart syndrome has been shown to be significantly associated with an increased risk of death and poor prognosis in patients with aSAH and ischemic stroke (27, 28).

We have demonstrated the association between BNP and 3-month prognosis in aSAH patients. However, the underlying mechanism remains elusive. Firstly, elevated BNP levels primarily reflect the compromised cardiac condition, and its association with neurological outcomes can be attributed to the impact of various newly developed cardiac disorders on the nervous system. The literature has reported on the predictive value of BNP in relation to cardiovascular events, such as atrial fibrillation, heart failure, and coronary heart disease (29, 30). Previous studies have also highlighted the link between atrial fibrillation and an increased incidence of stroke and mortality (31). Studies conducted in patients with cerebrovascular disease and coronary heart disease have indicated an elevated risk of widespread cerebral and carotid atherosclerosis (32). In addition to the common risk factors such as hyperlipidemia and smoking, vascular factors including thrombosis and endothelial dysfunction contribute to the risk. It is important to note that the lack of anticoagulant therapy guidelines in aSAH patients with concurrent cardiac events can potentially lead to bleeding risks (33).

Secondly, in patients with acute brain injury, particularly aSAH, a systemic inflammatory response and activation of endothelial cells can occur (34). This inflammatory response, along with the release of endothelin, can increase the burden on the heart and lead to dysfunction of cardiomyocytes. In response to cardiac injury, plasma BNP levels increase. Excessive inflammatory factors can also contribute to the disruption of the blood–brain barrier, allowing immune cells to infiltrate brain tissue. This infiltration can result in complications such as increased intracranial pressure and vasospasm, thereby increasing the risk of poor prognosis. In a prognostic model established by Runting Li et al., plasma white blood cell (WBC) count was found to have a significant correlation with patients’ outcomes (35), suggesting that inflammatory factors play a role in predicting prognosis in patients with acute brain injury.

Thirdly, the catecholamine surge hypothesis is widely accepted as the primary mechanism underlying brain-heart interactions. Studies, such as the one conducted by S. Naredi, have reported persistent sympathetic excitation and elevated levels of noradrenaline in patients with aSAH (36). The increased sympathetic activity can lead to direct myocardial injury and the subsequent release of excessive catecholamines, resulting in elevated BNP levels. Additionally, the enhanced sympathetic activity can affect baroreflex sensitivity (37), leading to increased blood pressure. In response to this, more BNP is secreted to lower blood pressure. Therefore, BNP can serve as an indicator of the severity and recoverability of autonomic nervous system damage. However, in our data, we did not observe a significant relationship between BNP and hypertension. This discrepancy may be attributed to differences in patients’ individual drug therapy, as well as the fact that the timing of blood pressure measurement may not precisely correspond to BNP levels. To investigate this mechanism further, continuous blood pressure monitoring should be conducted and compared longitudinally to observe the pattern of variability.

Finally, BNP may have a direct impact on the nervous system due to its vasodilatory and natriuretic functions. Elevated BNP levels can lead to a reduction in blood pressure, potentially resulting in insufficient cerebral perfusion pressure. This, in turn, increases the risk of ischemic cerebrovascular diseases such as delayed cerebral ischemia (DCI), which is a common poor prognostic outcome in patients with aSAH. Gurmeen Kaur reported a correlation between elevated BNP levels and the occurrence of DCI (17). Similar results were observed in our study. It is worth noting that BNP is not only secreted by cardiomyocytes but also by the hypothalamus. However, our data did not collect information regarding the localization of patients’ lesions, so it is unknown whether lesions involving the hypothalamus directly affect plasma BNP levels through its secretion. Further investigation would be required to explore this aspect in more detail.

There are several limitations in our study that should be acknowledged. Firstly, while the majority of our variables were derived from laboratory examinations, we lacked detailed cardiac examination and sufficient imaging data, particularly related to the structural aspects of the brain and heart. This limitation restricts our ability to fully assess how these structural factors may influence the prognosis of aSAH patients. Secondly, we did not collect detailed information about the medical treatments that patients received during their hospitalization, which could potentially affect their prognosis. Variations in treatment could introduce confounding factors that may influence the observed relationship between elevated BNP levels and poor prognosis. Thirdly, the underlying biological mechanisms linking elevated BNP to poor prognosis remain unclear. Further research is needed to elucidate the specific pathways through which BNP influences neurological outcomes. Finally, as a retrospective study, the level of evidence generated is lower compared to prospective studies. The reliance on pre-existing data introduces potential biases and limits the generalizability of the findings. Future prospective studies with larger sample sizes, more comprehensive data collection, and a focus on both clinical and imaging data would provide more robust insights into the relationship between BNP levels and prognosis in patients with aSAH.

## Conclusion

Elevated BNP stands as an independent risk factor of the 3-month prognosis in patients with aSAH. Although our models did not show significant improvement with the inclusion of BNP, it does not imply that monitoring BNP or considering its significance in clinical practice is futile. Elevated BNP serves as a robust indicator of cardiac injury and suggests a higher risk of cardiac events, which can indirectly impact neurological prognosis. Given these findings, we recommend that neurologists consult with cardiologists when BNP levels are elevated. Neurologists should work with cardiologists to assess cardiac function, identify any underlying cardiac conditions (e.g., heart failure, arrhythmias), and develop an individualized care plan. This collaboration can help optimize both cardiovascular and neurological management, reducing the risk of complications and improving overall prognosis. The efficacy of this therapeutic strategy should be further validated through prospective studies. BNP may not contribute significantly to prediction models, it still holds clinical relevance in guiding patient management and monitoring cardiac health in aSAH patients.

## Data Availability

The original contributions presented in the study are included in the article/Supplementary material, further inquiries can be directed to the corresponding authors.

## References

[1] Ingall T Asplund K Mähönen M Bonita R. A multinational comparison of subarachnoid hemorrhage epidemiology in the WHO MONICA stroke study. Stroke. (2000) 31:1054–61. doi: 10.1161/01.str.31.5.1054, PMID: 10797165

[2] Hop JW Rinkel GJ Algra A van Gijn J. Case-fatality rates and functional outcome after subarachnoid hemorrhage: a systematic review. Stroke. (1997) 28:660–4. doi: 10.1161/01.str.28.3.660, PMID: 9056628

[3] Kumar G Shahripour RB Harrigan MR. Vasospasm on transcranial Doppler is predictive of delayed cerebral ischemia in aneurysmal subarachnoid hemorrhage: a systematic review and meta-analysis. J Neurosurg. (2016) 124:1257–64. doi: 10.3171/2015.4.JNS15428, PMID: 26495942

[4] Rowland MJ Hadjipavlou G Kelly M Westbrook J Pattinson KT. Delayed cerebral ischaemia after subarachnoid haemorrhage: looking beyond vasospasm. Br J Anaesth. (2012) 109:315–29. doi: 10.1093/bja/aes264, PMID: 22879655

[5] Chen S Luo J Reis C Manaenko A Zhang J. Hydrocephalus after subarachnoid hemorrhage: pathophysiology, diagnosis, and treatment. Biomed Res Int. (2017) 2017:8584753–8. doi: 10.1155/2017/8584753, PMID: 28373987 PMC5360938

[6] Takahashi K Totsune K Sone M Ohneda M Murakami O Itoi K. Human brain natriuretic peptide-like immunoreactivity in human brain. Peptides. (1992) 13:121–3. doi: 10.1016/0196-9781(92)90149-w, PMID: 1535705

[7] Meaudre E Jego C Kenane N Montcriol A Boret H Goutorbe P. B-type natriuretic peptide release and left ventricular filling pressure assessed by echocardiographic study after subarachnoid hemorrhage: a prospective study in non-cardiac patients. Crit Care. (2009) 13:R76. doi: 10.1186/cc7891, PMID: 19454040 PMC2717439

[8] Lubien E DeMaria A Krishnaswamy P Clopton P Koon J Kazanegra R. Utility of B-natriuretic peptide in detecting diastolic dysfunction: comparison with Doppler velocity recordings. Circulation. (2002) 105:595–601. doi: 10.1161/hc0502.103010, PMID: 11827925

[9] Oppenheimer SM. Neurogenic cardiac effects of cerebrovascular disease. Curr Opin Neurol. (1994) 7:20–4. doi: 10.1097/00019052-199402000-00005, PMID: 8173672

[10] Sviri GE Soustiel JF Zaaroor M. Alteration in brain natriuretic peptide (BNP) plasma concentration following severe traumatic brain injury. Acta Neurochir. (2006) 148:529–33. doi: 10.1007/s00701-005-0666-4, PMID: 16322908

[11] Kirchhoff C Stegmaier J Bogner V Buhmann S Mussack T Kreimeier U. Intrathecal and systemic concentration of NT-pro BNP in patients with severe traumatic brain injury. J Neurotrauma. (2006) 23:943–9. doi: 10.1089/neu.2006.23.943, PMID: 16774478

[12] Oras J Grivans C Bartley A Rydenhag B Ricksten SE Seeman-Lodding H. Elevated high-sensitive troponin T on admission is an indicator of poor long-term outcome in patients with subarachnoid haemorrhage: a prospective observational study. Crit Care. (2016) 20:11. doi: 10.1186/s13054-015-1181-5, PMID: 26781032 PMC4717610

[13] Yip HK Sun CK Chang LT Chen MC Liou CW. Time course and prognostic value of plasma levels of N-terminal pro-brain natriuretic peptide in patients after ischemic stroke. Circ J. (2006) 70:447–52. doi: 10.1253/circj.70.447, PMID: 16565563

[14] Maruyama K Uchiyama S Shiga T Iijima M Ishizuka K Hoshino T. Brain natriuretic peptide is a powerful risk factor of outcome in stroke patients with atrial fibrillation. Cerebrovasc Dis Extra. (2017) 7:35–43. doi: 10.1159/000457808, PMID: 28253498 PMC5465753

[15] Maruyama K Shiga T Iijima M Moriya S Mizuno S Toi S. Brain natriuretic peptide in acute ischemic stroke. J Stroke Cerebrovasc Dis. (2014) 23:967–72. doi: 10.1016/j.jstrokecerebrovasdis.2013.08.003, PMID: 24119617

[16] Chihi M Darkwah Oppong M Quesada CM Dinger TF Gembruch O Pierscianek D. Role of brain natriuretic peptide in the prediction of early postoperative seizures following surgery for traumatic acute subdural hematoma: a prospective study. Neurol Ther. (2021) 10:847–63. doi: 10.1007/s40120-021-00269-w, PMID: 34342872 PMC8571437

[17] Kaur G Damodara N Feldstein E Dominguez J Huang KT Ogulnick JV. Relation between brain natriuretic peptide and delayed cerebral ischemia in patients with aneurysmalsubarachnoid hemorrhage. Clin Neurol Neurosurg. (2021) 211:107031. doi: 10.1016/j.clineuro.2021.107031, PMID: 34837820

[18] Taub PR Fields JD Wu AH Miss JC Lawton MT Smith WS. Elevated BNP is associated with vasospasm-independent cerebral infarction following aneurysmal subarachnoid hemorrhage. Neurocrit Care. (2011) 15:13–8. doi: 10.1007/s12028-011-9535-6, PMID: 21479679 PMC3133817

[19] Kishima H Mine T Ando T Yamada Y Tsuji M Ohmura T. Plasma brain natriuretic peptide level on admission predicts long-term outcome in patients with non-traumatic subarachnoid hemorrhage. J Clin Neurosci. (2020) 79:7–11. doi: 10.1016/j.jocn.2020.07.031, PMID: 33070921

[20] Connolly ES Jr Rabinstein AA Carhuapoma JR Derdeyn CP Dion J Higashida RT. Council on cardiovascular radiology and intervention; council on cardiovascular nursing; council on cardiovascular surgery and anesthesia; council on clinical cardiology. Guidelines for the management of aneurysmal subarachnoid hemorrhage: a guideline for healthcare professionals from the American Heart Association/american Stroke Association. Stroke. (2012) 43:1711–37. doi: 10.1161/STR.0b013e3182587839 22556195

[21] Maron BJ Tholakanahalli VN Zenovich AG Casey SA Duprez D Aeppli DM. Usefulness of B-type natriuretic peptide assay in the assessment of symptomatic state in hypertrophic cardiomyopathy. Circulation. (2004) 109:984–9. doi: 10.1161/01.CIR.0000117098.75727.D8, PMID: 14967727

[22] James ML Blessing R Phillips-Bute BG Bennett E Laskowitz DT. S100B and brain natriuretic peptide predict functional neurological outcome after intracerebral haemorrhage. Biomarkers. (2009) 14:388–94. doi: 10.1080/13547500903015784, PMID: 19505208 PMC2731822

[23] McAteer A Hravnak M Chang Y Crago EA Gallek MJ Yousef KM. The relationships between BNP and Neurocardiac injury severity, noninvasive cardiac output, and outcomes after aneurysmal subarachnoid hemorrhage. Biol Res Nurs. (2017) 19:531–7. doi: 10.1177/1099800417711584, PMID: 28627225 PMC5494000

[24] Prosser J Mac Gregor L Lees KR Diener HC Hacke W Davis S. VISTA investigators. Risk factors of early cardiac morbidity and mortality after ischemic stroke. Stroke. (2007) 38:2295–302. doi: 10.1161/STROKEAHA.106.471813, PMID: 17569877

[25] Tahsili-Fahadan P Geocadin RG. Heart-brain Axis: effects of neurologic injury on cardiovascular function. Circ Res. (2017) 120:559–72. doi: 10.1161/CIRCRESAHA.116.308446 28154104

[26] Scheitz JF Nolte CH Doehner W Hachinski V Endres M. Stroke-heart syndrome: clinical presentation and underlying mechanisms. Lancet Neurol. (2018) 17:1109–20. doi: 10.1016/S1474-4422(18)30336-3, PMID: 30509695

[27] van der Bilt IA Hasan D Vandertop WP Wilde AA Algra A Visser FC. Impact of cardiac complications on outcome after aneurysmal subarachnoid hemorrhage: a meta-analysis. Neurology. (2009) 72:635–42. doi: 10.1212/01.wnl.0000342471.07290.07, PMID: 19221297

[28] Sörös P Hachinski V. Cardiovascular and neurological causes of sudden death after ischaemic stroke. Lancet Neurol. (2012) 11:179–88. doi: 10.1016/S1474-4422(11)70291-5, PMID: 22265213

[29] Wang TJ Larson MG Levy D Benjamin EJ Leip EP Omland T. Plasma natriuretic peptide levels and the risk of cardiovascular events and death. N Engl J Med. (2004) 350:655–63. doi: 10.1056/NEJMoa031994 14960742

[30] Puy L Forman R Cordonnier C Sheth KN. Protecting the brain, from the heart: safely mitigating the consequences of thrombosis in intracerebral hemorrhage survivors with atrial fibrillation. Stroke. (2022) 53:2152–60. doi: 10.1161/STROKEAHA.122.036888, PMID: 35759545

[31] Oremus M Raina PS Santaguida P Balion CM McQueen MJ McKelvie R. A systematic review of BNP as a risk factor of prognosis in persons with coronary artery disease. Clin Biochem. (2008) 41:260–5. doi: 10.1016/j.clinbiochem.2007.09.011, PMID: 17949703

[32] Migdady I Russman A Buletko AB. Atrial fibrillation and ischemic stroke: a clinical review. Semin Neurol. (2021) 41:348–64. doi: 10.1055/s-0041-1726332, PMID: 33851396

[33] Kong Q Ma X Wang C Feng W Ovbiagele B Zhang Y. Patients with acute ischemic cerebrovascular disease with coronary artery stenosis have more diffused Cervicocephalic atherosclerosis. J Atheroscler Thromb. (2019) 26:792–804. doi: 10.5551/jat.47464, PMID: 30726790 PMC6753244

[34] Saand AR Yu F Chen J Chou SH. Systemic inflammation in hemorrhagic strokes - a novel neurological sign and therapeutic target? J Cereb Blood Flow Metab. (2019) 39:959–88. doi: 10.1177/0271678X19841443, PMID: 30961425 PMC6547186

[35] Li R Lin F Chen Y Lu J Han H Ma L. A 90-day prognostic model based on the early brain injury indicators after aneurysmal subarachnoid hemorrhage: the TAPS score. Transl Stroke Res. (2023) 14:200–10. doi: 10.1007/s12975-022-01033-4, PMID: 35567655

[36] Naredi S Lambert G Edén E Zäll S Runnerstam M Rydenhag B. Increased sympathetic nervous activity in patients with nontraumatic subarachnoid hemorrhage. Stroke. (2000) 31:901–6. doi: 10.1161/01.str.31.4.901, PMID: 10753996

[37] Sykora M Steiner T Rocco A Turcani P Hacke W Diedler J. Baroreflex sensitivity to predict malignant middle cerebral artery infarction. Stroke. (2012) 43:714–9. doi: 10.1161/STROKEAHA.111.632778, PMID: 22223241

