# Supplementary material for: Predicting prognosis using stroke-heart indicator: brain natriuretic peptide in patients with aneurysmal subarachnoid hemorrhage
Source: Front Neurol. 2025 Jan 22;16:1510235. doi: 10.3389/fneur.2025.1510235 (PMC11794076; doi:10.3389/fneur.2025.1510235)
Supplement: Supplementary file 1 [file Data_Sheet_1.pdf]

Positive if Greater Than or Equal To	Sensitivity	1 - Specificity		Youden index
61.3	0.632	0.444		0.188
61.15	0.632	0.445		0.187
56.1	0.661	0.476		0.185
61.05	0.632	0.447		0.185
63.95	0.62	0.435		0.185
60.95	0.632	0.448		0.184
62	0.626	0.442		0.184
63.55	0.62	0.436		0.184
60.85	0.632	0.449		0.183
61.75	0.626	0.443		0.183
52.65	0.684	0.502		0.182
54.55	0.673	0.491		0.182
56.7	0.655	0.473		0.182
61.5	0.626	0.444		0.182
63.1	0.62	0.438		0.182
55.8	0.661	0.48		0.181
56.55	0.655	0.474		0.181
60.2	0.637	0.456		0.181
62.85	0.62	0.439		0.181
60.75	0.632	0.452		0.18
62.5	0.62	0.44		0.18
52.55	0.684	0.505		0.179
53.55	0.678	0.499		0.179
54.35	0.673	0.494		0.179
55.6	0.661	0.482		0.179
56.4	0.655	0.476		0.179
60.65	0.632	0.453		0.179
52.25	0.684	0.506		0.178
54.9	0.667	0.489		0.178
59.95	0.637	0.459		0.178
62.2	0.62	0.442		0.178
52.95	0.678	0.501		0.177
54.75	0.667	0.49		0.177
57.3	0.649	0.472		0.177
59.65	0.637	0.46		0.177
60.55	0.632	0.455		0.177
54.2	0.673	0.497		0.176
55.4	0.661	0.485		0.176
57	0.649	0.473		0.176
59.35	0.637	0.461		0.176
60.4	0.632	0.456		0.176
51.9	0.684	0.509		0.175
54.05	0.673	0.498		0.175
55.15	0.661	0.486		0.175
53.95	0.673	0.499		0.174
59.05	0.637	0.463		0.174
64.1	0.608	0.434		0.174
51.75	0.684	0.511		0.173
58.8	0.637	0.464		0.173
58.65	0.637	0.465		0.172
57.55	0.643	0.472		0.171
58.3	0.637	0.466		0.171
51.65	0.684	0.514		0.17
48.1	0.713	0.543		0.17
57.95	0.637	0.468		0.169
51.35	0.684	0.516		0.168
57.85	0.637	0.469		0.168
47.85	0.713	0.545		0.168
57.75	0.637	0.47		0.167
51	0.684	0.519		0.165
47.75	0.713	0.548		0.165
47.65	0.713	0.549		0.164
44.85	0.731	0.569		0.162
50.8	0.684	0.522		0.162
64.3	0.596	0.434		0.162
47.5	0.713	0.551		0.162
44.75	0.731	0.57		0.161
45.85	0.725	0.564		0.161
50.55	0.684	0.523		0.161
47.35	0.713	0.552		0.161
45.4	0.725	0.565		0.16
50.35	0.684	0.524		0.16
47.25	0.713	0.553		0.16
44.05	0.737	0.578		0.159
44.6	0.731	0.572		0.159
45.1	0.725	0.566		0.159
48.35	0.702	0.543		0.159
44.4	0.731	0.573		0.158
47.1	0.713	0.555		0.158
43.85	0.737	0.58		0.157
50.25	0.684	0.527		0.157
64.45	0.591	0.434		0.157
46.95	0.713	0.556		0.157
42.15	0.749	0.593		0.156
43.65	0.737	0.581		0.156
44.95	0.725	0.569		0.156
50.1	0.684	0.528		0.156
46.8	0.713	0.557		0.156
49.45	0.69	0.534		0.156
42	0.749	0.594		0.155
43.55	0.737	0.582		0.155
44.25	0.731	0.576		0.155
46.15	0.719	0.564		0.155
46.6	0.713	0.558		0.155
43.45	0.737	0.583		0.154
49.9	0.684	0.53		0.154
64.75	0.585	0.431		0.154
49.2	0.69	0.536		0.154
42.45	0.743	0.59		0.153
44.15	0.731	0.578		0.153
49.7	0.684	0.531		0.153
48.45	0.696	0.543		0.153
49.05	0.69	0.537		0.153
41.65	0.749	0.597		0.152
42.25	0.743	0.591		0.152
43.35	0.737	0.585		0.152
46.45	0.713	0.561		0.152
41.2	0.749	0.598		0.151
43.25	0.737	0.586		0.151
64.6	0.585	0.434		0.151
46.35	0.713	0.562		0.151
48.9	0.69	0.539		0.151
42.9	0.737	0.587		0.15
135.35	0.351	0.201		0.15
48.75	0.69	0.54		0.15
46.25	0.713	0.564		0.149
64.95	0.579	0.43		0.149
135.15	0.351	0.202		0.149
48.65	0.69	0.541		0.149
40.9	0.749	0.601		0.148
133.35	0.357	0.209		0.148
68.25	0.561	0.414		0.147
40.75	0.749	0.602		0.147
132.6	0.357	0.21		0.147
134.95	0.351	0.204		0.147
48.55	0.69	0.543		0.147
67.75	0.561	0.415		0.146
40.55	0.749	0.603		0.146
137.85	0.339	0.193		0.146
134.55	0.351	0.205		0.146
40.35	0.749	0.604		0.145
137.35	0.339	0.194		0.145
132	0.357	0.212		0.145
134.1	0.351	0.206		0.145
67.25	0.567	0.422		0.145
135.7	0.345	0.2		0.145
131.45	0.357	0.213		0.144
66.9	0.567	0.423		0.144
67.45	0.561	0.418		0.143
68.55	0.556	0.413		0.143
40.25	0.749	0.606		0.143
130.95	0.357	0.214		0.143
65.15	0.573	0.43		0.143
66.25	0.567	0.424		0.143
40.1	0.749	0.607		0.142
136.8	0.339	0.197		0.142
138.95	0.333	0.191		0.142
141.5	0.327	0.185		0.142
144.4	0.322	0.18		0.142
133.85	0.351	0.209		0.142
102.05	0.421	0.279		0.142
67.35	0.561	0.42		0.141
136.3	0.339	0.198		0.141
138.85	0.333	0.192		0.141
143.3	0.322	0.181		0.141
130.2	0.357	0.216		0.141
65.75	0.567	0.426		0.141
101.35	0.421	0.28		0.141
138.4	0.333	0.193		0.14
128.85	0.357	0.217		0.14
65.45	0.567	0.427		0.14
101.05	0.421	0.281		0.14
72.1	0.532	0.393		0.139
140.5	0.327	0.188		0.139
145.45	0.316	0.177		0.139
128.05	0.357	0.218		0.139
65.25	0.567	0.428		0.139
39.95	0.749	0.611		0.138
71.9	0.532	0.394		0.138
100.8	0.421	0.283		0.138
139.5	0.327	0.189		0.138
142.5	0.322	0.184		0.138
151.05	0.31	0.172		0.138
68.75	0.55	0.413		0.137
39.85	0.749	0.612		0.137
98.35	0.427	0.29		0.137
100.55	0.421	0.284		0.137
142.2	0.322	0.185		0.137
145.1	0.316	0.179		0.137
149.8	0.31	0.173		0.137
71.6	0.532	0.396		0.136
72.85	0.526	0.39		0.136
100.45	0.421	0.285		0.136
144.9	0.316	0.18		0.136
127.55	0.357	0.221		0.136
69.1	0.544	0.409		0.135
39.75	0.749	0.614		0.135
71.35	0.532	0.397		0.135
97.8	0.427	0.292		0.135
100.1	0.421	0.286		0.135
110.25	0.386	0.251		0.135
147.85	0.31	0.175		0.135
127.05	0.357	0.222		0.135
68.95	0.544	0.41		0.134
72.55	0.526	0.392		0.134
109.8	0.386	0.252		0.134
145.95	0.31	0.176		0.134
125.2	0.363	0.229		0.134
126.9	0.357	0.223		0.134
39.55	0.749	0.616		0.133
71.25	0.532	0.399		0.133
72.25	0.526	0.393		0.133
97.65	0.427	0.294		0.133
99.75	0.421	0.288		0.133
111.4	0.38	0.247		0.133
124.1	0.363	0.23		0.133
69.95	0.538	0.406		0.132
81.2	0.485	0.353		0.132
99.6	0.421	0.289		0.132
109.4	0.386	0.254		0.132
152.5	0.304	0.172		0.132
122.8	0.363	0.231		0.132
126.65	0.357	0.225		0.132
69.6	0.538	0.407		0.131
39.35	0.749	0.618		0.131
71.1	0.532	0.401		0.131
86.95	0.462	0.331		0.131
88.4	0.456	0.325		0.131
97.45	0.427	0.296		0.131
99.15	0.421	0.29		0.131
104.15	0.404	0.273		0.131
109.15	0.386	0.255		0.131
126.3	0.357	0.226		0.131
39.25	0.749	0.619		0.13
70.95	0.532	0.402		0.13
72.95	0.52	0.39		0.13
80.8	0.485	0.355		0.13
86.75	0.462	0.332		0.13
87.85	0.456	0.326		0.13
93.65	0.439	0.309		0.13
97.2	0.427	0.297		0.13
109.05	0.386	0.256		0.13
110.75	0.38	0.25		0.13
121.1	0.363	0.233		0.13
126.05	0.357	0.227		0.13
102.55	0.409	0.279		0.13
69.3	0.538	0.409		0.129
37.1	0.766	0.637		0.129
38.05	0.76	0.631		0.129
39.15	0.749	0.62		0.129
70.7	0.532	0.403		0.129
73.4	0.515	0.386		0.129
78.05	0.497	0.368		0.129
84.9	0.468	0.339		0.129
87.55	0.456	0.327		0.129
92.95	0.439	0.31		0.129
96.75	0.427	0.298		0.129
103.85	0.404	0.275		0.129
105.45	0.398	0.269		0.129
106.8	0.392	0.263		0.129
120.05	0.363	0.234		0.129
37.95	0.76	0.632		0.128
77.95	0.497	0.369		0.128
80.5	0.485	0.357		0.128
84.7	0.468	0.34		0.128
86.6	0.462	0.334		0.128
89.55	0.45	0.322		0.128
91.95	0.439	0.311		0.128
103.35	0.404	0.276		0.128
106.4	0.392	0.264		0.128
108.95	0.386	0.258		0.128
112.9	0.374	0.246		0.128
119.45	0.363	0.235		0.128
37.75	0.76	0.633		0.127
39.05	0.749	0.622		0.127
70.3	0.532	0.405		0.127
73.25	0.515	0.388		0.127
79.1	0.491	0.364		0.127
86.25	0.462	0.335		0.127
87.35	0.456	0.329		0.127
89.2	0.45	0.323		0.127
94.35	0.433	0.306		0.127
96.2	0.427	0.3		0.127
103.05	0.404	0.277		0.127
106.25	0.392	0.265		0.127
108.75	0.386	0.259		0.127
112.3	0.374	0.247		0.127
36.55	0.766	0.64		0.126
73.15	0.515	0.389		0.126
77.85	0.497	0.371		0.126
78.65	0.491	0.365		0.126
80.35	0.485	0.359		0.126
84.55	0.468	0.342		0.126
85.65	0.462	0.336		0.126
87.2	0.456	0.33		0.126
91.3	0.439	0.313		0.126
94.25	0.433	0.307		0.126
95.4	0.427	0.301		0.126
105.3	0.398	0.272		0.126
108.3	0.386	0.26		0.126
115.55	0.368	0.242		0.126
119.15	0.363	0.237		0.126
153.4	0.298	0.172		0.126
37.55	0.76	0.635		0.125
38.25	0.754	0.629		0.125
73.05	0.515	0.39		0.125
75.5	0.503	0.378		0.125
77.6	0.497	0.372		0.125
80.2	0.485	0.36		0.125
84.25	0.468	0.343		0.125
88.85	0.45	0.325		0.125
91.15	0.439	0.314		0.125
94.75	0.427	0.302		0.125
102.8	0.404	0.279		0.125
104.7	0.398	0.273		0.125
105.85	0.392	0.267		0.125
107.6	0.386	0.261		0.125
114.55	0.368	0.243		0.125
119.05	0.363	0.238		0.125
83.95	0.468	0.344		0.124
38.95	0.749	0.625		0.124
73.9	0.509	0.385		0.124
77.35	0.497	0.373		0.124
78.25	0.491	0.367		0.124
80	0.485	0.361		0.124
85.15	0.462	0.338		0.124
94.05	0.433	0.309		0.124
114.25	0.368	0.244		0.124
118.65	0.363	0.239		0.124
156.05	0.292	0.168		0.124
36.35	0.766	0.643		0.123
75.25	0.503	0.38		0.123
78.15	0.491	0.368		0.123
82.2	0.474	0.351		0.123
107.15	0.386	0.263		0.123
118.15	0.363	0.24		0.123
83.8	0.468	0.346		0.122
38.7	0.749	0.627		0.122
75.15	0.503	0.381		0.122
77.05	0.497	0.375		0.122
79.8	0.485	0.363		0.122
89.75	0.444	0.322		0.122
91.05	0.439	0.317		0.122
94.6	0.427	0.305		0.122
113.5	0.368	0.246		0.122
155.15	0.292	0.17		0.122
83.45	0.468	0.347		0.121
36.25	0.766	0.645		0.121
38.45	0.749	0.628		0.121
75	0.503	0.382		0.121
76.45	0.497	0.376		0.121
79.45	0.485	0.364		0.121
81.75	0.474	0.353		0.121
90.85	0.439	0.318		0.121
94.45	0.427	0.306		0.121
117.25	0.363	0.242		0.121
154.85	0.292	0.171		0.121
83	0.468	0.348		0.12
28.45	0.848	0.728		0.12
75.9	0.497	0.377		0.12
90.5	0.439	0.319		0.12
154.25	0.292	0.172		0.12
28.35	0.848	0.729		0.119
36.15	0.766	0.647		0.119
74.6	0.503	0.384		0.119
156.9	0.287	0.168		0.119
36.05	0.766	0.648		0.118
90.15	0.439	0.321		0.118
82.55	0.468	0.351		0.117
89.95	0.439	0.322		0.117
28.15	0.848	0.732		0.116
28.55	0.842	0.727		0.115
158.05	0.281	0.167		0.114
157.45	0.281	0.168		0.113
27.9	0.848	0.735		0.113
35.9	0.766	0.653		0.113
35.7	0.766	0.654		0.112
27.65	0.848	0.737		0.111
28.65	0.836	0.725		0.111
162.1	0.269	0.159		0.11
33.6	0.784	0.674		0.11
35.55	0.766	0.656		0.11
159.35	0.275	0.166		0.109
161.95	0.269	0.16		0.109
27.45	0.848	0.739		0.109
33.2	0.784	0.675		0.109
35.45	0.766	0.657		0.109
158.65	0.275	0.167		0.108
27.3	0.848	0.74		0.108
29.85	0.819	0.711		0.108
34.1	0.778	0.67		0.108
161.45	0.269	0.162		0.107
27.05	0.848	0.741		0.107
33.95	0.778	0.671		0.107
160.95	0.269	0.163		0.106
28.75	0.83	0.724		0.106
28.95	0.825	0.719		0.106
33.05	0.784	0.678		0.106
160.3	0.269	0.164		0.105
164.65	0.263	0.158		0.105
28.85	0.825	0.72		0.105
29.65	0.819	0.714		0.105
35.2	0.766	0.661		0.105
162.7	0.263	0.159		0.104
26.8	0.848	0.744		0.104
29.45	0.819	0.715		0.104
34.95	0.766	0.662		0.104
159.65	0.269	0.166		0.103
26.6	0.848	0.745		0.103
30.05	0.813	0.71		0.103
32.95	0.784	0.681		0.103
34.45	0.772	0.669		0.103
26.45	0.848	0.746		0.102
29.25	0.819	0.717		0.102
32.85	0.784	0.682		0.102
34.25	0.772	0.67		0.102
34.85	0.766	0.664		0.102
167.1	0.257	0.156		0.101
32.75	0.784	0.683		0.101
171.15	0.251	0.151		0.1
176	0.246	0.146		0.1
26.35	0.848	0.748		0.1
29.1	0.819	0.719		0.1
30.15	0.807	0.708		0.099
166.4	0.257	0.158		0.099
170.95	0.251	0.152		0.099
174.95	0.246	0.147		0.099
26.2	0.848	0.749		0.099
173.9	0.246	0.148		0.098
34.75	0.766	0.668		0.098
32.6	0.784	0.687		0.097
34.65	0.766	0.669		0.097
170.45	0.251	0.155		0.096
172.6	0.246	0.15		0.096
26.05	0.848	0.752		0.096
32.45	0.784	0.689		0.095
168.75	0.251	0.156		0.095
171.55	0.246	0.151		0.095
25.75	0.848	0.753		0.095
30.3	0.801	0.707		0.094
177.2	0.24	0.146		0.094
25.35	0.848	0.754		0.094
32.3	0.784	0.691		0.093
31.05	0.795	0.703		0.092
30.75	0.795	0.704		0.091
31.8	0.789	0.698		0.091
24.5	0.854	0.763		0.091
25.15	0.848	0.757		0.091
31.5	0.789	0.699		0.09
32.1	0.784	0.694		0.09
30.55	0.795	0.706		0.089
31.25	0.789	0.7		0.089
178.5	0.234	0.145		0.089
30.45	0.795	0.707		0.088
177.95	0.234	0.146		0.088
268.55	0.152	0.064		0.088
24.3	0.854	0.766		0.088
25.05	0.848	0.76		0.088
24.15	0.854	0.767		0.087
24.9	0.848	0.761		0.087
31.95	0.784	0.698		0.086
180.2	0.228	0.142		0.086
267.25	0.152	0.066		0.086
179.2	0.228	0.143		0.085
23.95	0.854	0.769		0.085
24.7	0.848	0.763		0.085
188.15	0.211	0.127		0.084
264.45	0.152	0.068		0.084
274.05	0.146	0.062		0.084
23.75	0.854	0.77		0.084
271.25	0.146	0.063		0.083
21.8	0.877	0.794		0.083
23.55	0.854	0.771		0.083
253.3	0.158	0.076		0.082
187.8	0.211	0.129		0.082
261.2	0.152	0.07		0.082
269.55	0.146	0.064		0.082
259.4	0.152	0.071		0.081
187.1	0.211	0.13		0.081
21.6	0.877	0.796		0.081
23.3	0.854	0.773		0.081
181.45	0.222	0.142		0.08
252.4	0.158	0.078		0.08
257.65	0.152	0.072		0.08
186.2	0.211	0.131		0.08
251.7	0.158	0.079		0.079
190.15	0.205	0.126		0.079
21.95	0.871	0.792		0.079
19.75	0.895	0.817		0.078
277.05	0.14	0.062		0.078
251.3	0.158	0.08		0.078
255.25	0.152	0.074		0.078
182.8	0.216	0.138		0.078
185.5	0.211	0.133		0.078
188.7	0.205	0.127		0.078
21.45	0.877	0.799		0.078
250.5	0.158	0.081		0.077
254.25	0.152	0.075		0.077
182.3	0.216	0.139		0.077
185.05	0.211	0.134		0.077
20.7	0.883	0.806		0.077
21.3	0.877	0.8		0.077
22.05	0.865	0.788		0.077
23.15	0.854	0.777		0.077
283.15	0.135	0.059		0.076
199.9	0.193	0.117		0.076
253.8	0.152	0.076		0.076
184.75	0.211	0.135		0.076
23.05	0.854	0.778		0.076
19.65	0.895	0.82		0.075
181.95	0.216	0.141		0.075
280.2	0.135	0.06		0.075
249.5	0.158	0.083		0.075
20.55	0.883	0.808		0.075
22.9	0.854	0.779		0.075
20.1	0.889	0.815		0.074
191.3	0.199	0.125		0.074
223.15	0.17	0.096		0.074
201.05	0.187	0.113		0.074
247.8	0.158	0.084		0.074
184.05	0.211	0.137		0.074
21	0.877	0.803		0.074
19.95	0.889	0.816		0.073
199.25	0.193	0.12		0.073
221.8	0.17	0.097		0.073
279.05	0.135	0.062		0.073
200.45	0.187	0.114		0.073
246.25	0.158	0.085		0.073
183.25	0.211	0.138		0.073
22.75	0.854	0.781		0.073
19.5	0.895	0.823		0.072
19.85	0.889	0.817		0.072
197.8	0.193	0.121		0.072
20.4	0.883	0.811		0.072
22.2	0.86	0.788		0.072
22.65	0.854	0.782		0.072
195.2	0.193	0.122		0.071
218.85	0.17	0.099		0.071
245.25	0.158	0.087		0.071
200.3	0.187	0.116		0.071
206.95	0.175	0.104		0.071
22.55	0.854	0.783		0.071
19.35	0.895	0.825		0.07
20.25	0.883	0.813		0.07
216	0.17	0.1		0.07
243.8	0.158	0.088		0.07
286.15	0.129	0.059		0.07
200.1	0.187	0.117		0.07
206.05	0.175	0.105		0.07
22.45	0.854	0.784		0.07
15.05	0.936	0.867		0.069
16.45	0.924	0.855		0.069
193	0.193	0.124		0.069
215.2	0.17	0.101		0.069
241.95	0.158	0.089		0.069
205.7	0.175	0.106		0.069
15.45	0.93	0.862		0.068
214.4	0.17	0.102		0.068
191.95	0.193	0.125		0.068
224.35	0.164	0.096		0.068
201.8	0.181	0.113		0.068
15.35	0.93	0.863		0.067
16.2	0.924	0.857		0.067
19.25	0.895	0.828		0.067
240.45	0.158	0.091		0.067
204.9	0.175	0.108		0.067
22.35	0.854	0.787		0.067
16.05	0.924	0.858		0.066
16.75	0.918	0.852		0.066
19.15	0.895	0.829		0.066
210.9	0.17	0.104		0.066
238.9	0.158	0.092		0.066
204.2	0.175	0.109		0.066
14.9	0.936	0.871		0.065
15.25	0.93	0.865		0.065
15.85	0.924	0.859		0.065
19.05	0.895	0.83		0.065
235.7	0.158	0.093		0.065
292.9	0.123	0.058		0.065
203.65	0.175	0.11		0.065
15.15	0.93	0.866		0.064
16.65	0.918	0.854		0.064
17.9	0.906	0.842		0.064
18.35	0.901	0.837		0.064
288.15	0.123	0.059		0.064
351.05	0.111	0.047		0.064
17.15	0.912	0.849		0.063
229.75	0.158	0.095		0.063
321.7	0.117	0.054		0.063
203.15	0.175	0.112		0.063
14.75	0.936	0.874		0.062
15.6	0.924	0.862		0.062
17.05	0.912	0.85		0.062
17.7	0.906	0.844		0.062
18.95	0.895	0.833		0.062
315.25	0.117	0.055		0.062
225.3	0.158	0.096		0.062
340.6	0.111	0.049		0.062
413.35	0.105	0.043		0.062
202.5	0.175	0.113		0.062
14.6	0.936	0.875		0.061
18.25	0.901	0.84		0.061
18.7	0.895	0.834		0.061
333.55	0.111	0.05		0.061
14.45	0.936	0.876		0.06
16.9	0.912	0.852		0.06
17.5	0.906	0.846		0.06
18.1	0.901	0.841		0.06
308.5	0.117	0.057		0.06
330.75	0.111	0.051		0.06
405.3	0.105	0.045		0.06
301.9	0.117	0.058		0.059
379.35	0.105	0.046		0.059
17.3	0.906	0.848		0.058
18.45	0.895	0.837		0.058
326.95	0.111	0.053		0.058
358.45	0.105	0.047		0.058
14.3	0.936	0.879		0.057
323.4	0.111	0.054		0.057
413.6	0.099	0.043		0.056
436.7	0.094	0.039		0.055
14.15	0.936	0.882		0.054
14.05	0.936	0.883		0.053
432	0.094	0.041		0.053
466.8	0.088	0.035		0.053
13.9	0.936	0.884		0.052
422.5	0.094	0.042		0.052
414.4	0.094	0.043		0.051
458.65	0.088	0.037		0.051
13.75	0.936	0.886		0.05
449.15	0.088	0.038		0.05
13.65	0.936	0.887		0.049
441.4	0.088	0.039		0.049
13.55	0.936	0.888		0.048
479.55	0.082	0.035		0.047
13.4	0.936	0.89		0.046
13.25	0.936	0.892		0.044
11.95	0.947	0.904		0.043
13.1	0.936	0.894		0.042
11.8	0.947	0.905		0.042
12.45	0.942	0.9		0.042
12.95	0.936	0.895		0.041
12.3	0.942	0.901		0.041
11.65	0.947	0.907		0.04
11.55	0.947	0.908		0.039
12.15	0.942	0.903		0.039
12.8	0.936	0.898		0.038
634.9	0.064	0.026		0.038
12.05	0.942	0.904		0.038
12.6	0.936	0.899		0.037
629.05	0.064	0.028		0.036
509.45	0.07	0.035		0.035
620.4	0.064	0.029		0.035
11.45	0.947	0.912		0.035
599.9	0.064	0.03		0.034
740.4	0.058	0.024		0.034
715.4	0.058	0.025		0.033
1014.05	0.047	0.014		0.033
566.15	0.064	0.032		0.032
671	0.058	0.026		0.032
11.35	0.947	0.915		0.032
543	0.064	0.033		0.031
802.1	0.053	0.022		0.031
1002.2	0.047	0.016		0.031
11.2	0.947	0.916		0.031
534.65	0.064	0.034		0.03
946.65	0.047	0.017		0.03
11.05	0.947	0.917		0.03
869	0.047	0.018		0.029
1229.4	0.041	0.012		0.029
532.15	0.064	0.035		0.029
776	0.053	0.024		0.029
10.65	0.953	0.924		0.029
1091.5	0.041	0.013		0.028
10.55	0.953	0.925		0.028
10.95	0.947	0.919		0.028
1024.35	0.041	0.014		0.027
830.95	0.047	0.02		0.027
820.3	0.047	0.021		0.026
10.85	0.947	0.921		0.026
814.45	0.047	0.022		0.025
10.35	0.953	0.928		0.025
1306.95	0.035	0.012		0.023
10.15	0.953	0.93		0.023
10.75	0.947	0.924		0.023
10.05	0.953	0.933		0.02
9.3	0.959	0.94		0.019
9.8	0.953	0.934		0.019
1349.2	0.029	0.011		0.018
8.95	0.959	0.942		0.017
1311.45	0.029	0.012		0.017
7.9	0.965	0.95		0.015
7.75	0.965	0.951		0.014
8.7	0.959	0.945		0.014
8.5	0.959	0.946		0.013
9.55	0.953	0.94		0.013
7.65	0.965	0.953		0.012
1431.3	0.023	0.011		0.012
7.55	0.965	0.955		0.01
8.2	0.959	0.95		0.009
7.45	0.965	0.957		0.008
7.1	0.965	0.958		0.007
1521.6	0.018	0.011		0.007
6.75	0.965	0.961		0.004
1794.05	0.012	0.008		0.004
1723.4	0.012	0.009		0.003
6.65	0.965	0.963		0.002
2635.6	0.006	0.004		0.002
6.05	0.971	0.97		0.001
1620.25	0.012	0.011		0.001
2493.9	0.006	0.005		0.001
-1	1	1		0
6.55	0.965	0.965		0
4920.6	0	0		0
2335.25	0.006	0.007		-0.001
3918.55	0	0.001		-0.001
5.9	0.971	0.972		-0.001
6.35	0.965	0.966		-0.001
2040.5	0.006	0.008		-0.002
6.15	0.965	0.967		-0.002
2827.5	0	0.003		-0.003
2713.35	0	0.004		-0.004
5.5	0.971	0.975		-0.004
0.635	0.994	0.999		-0.005
2.35	0.988	0.993		-0.005
3.75	0.982	0.987		-0.005
5.15	0.971	0.976		-0.005
0.035	0.994	1		-0.006
4.3	0.977	0.983		-0.006
2.05	0.988	0.995		-0.007
3.5	0.982	0.989		-0.007
3.95	0.977	0.984		-0.007
5.05	0.971	0.978		-0.007
1.9	0.988	0.996		-0.008
4.95	0.971	0.979		-0.008
1.6	0.988	0.997		-0.009
3.15	0.982	0.991		-0.009
3.85	0.977	0.986		-0.009
4.8	0.971	0.98		-0.009
2.85	0.982	0.992		-0.01
1.3	0.988	0.999		-0.011
2.65	0.982	0.993		-0.011
4.65	0.971	0.982		-0.011

	Estimate	Std.Error	Lower	Upper
NRI	0.011104195	0.013233431	-0.014201940	0.03837042
NRI+	0.005847953	0.012899935	-0.017804311	0.03125122
NRI-	0.005256242	0.002622588	0.001291156	0.01065957
Pr(Up|Case)	0.017543860	0.009781655	0.000000000	0.03921599
Pr(Down|Case)	0.011695906	0.008172459	0.000000000	0.03021176
Pr(Down|Ctrl)	0.005256242	0.002622588	0.001291156	0.01065957
Pr(Up|Ctrl)	0.000000000	0.000000000	0.000000000	0.00000000

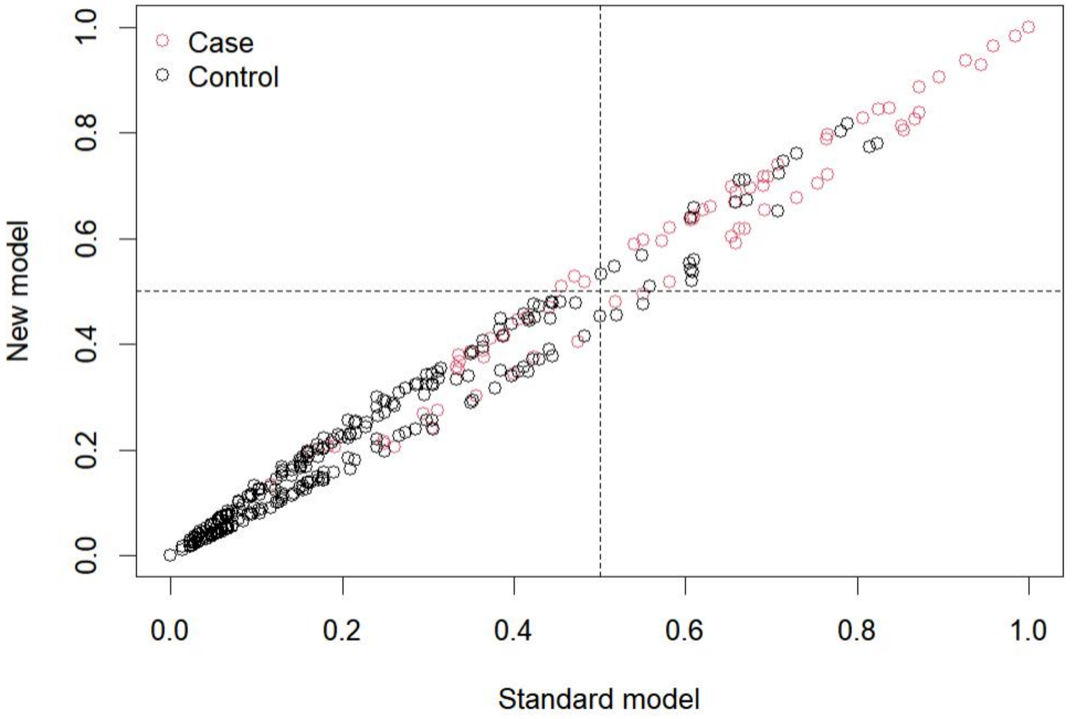

NRI=0.0111 (-0.0142-0.0384),P=0.401

Model 1 AUC: 0.828
> cat("Model 2 AUC:", round(auc(roc2), 3), "\n")
Model 2 AUC: 0.828
> delong_test <- roc.test(roc1, roc2, method = "delong")
> cat("DeLong Test P-Value:", delong_test$p.value, "\n")
DeLong Test P-Value: 0.5968959

P＞0.05，It is considered that the AUC of both models A and B may not be significant
